# Apolipoprotein E ɛ4–related effects on cognition are limited to the Alzheimer’s disease spectrum

**DOI:** 10.1007/s11357-021-00450-x

**Published:** 2021-09-30

**Authors:** Alberto Fernández, Lucía Vaquero, Ricardo Bajo, Pilar Zuluaga, ADNI–Alzheimer’s Disease Neuroimaging Initiative, Michael W. Weiner, Andrew J. Saykin, John Q. Trojanowski, Leslie Shaw, Arthur W. Toga, Laurel Beckett, Clifford R. Jack, Paul Aisen, Ronald C. Petersen, John C. Morris, William Jagust

**Affiliations:** 1 Legal Medicine, Psychiatry, and Pathology Department, Faculty of Medicine, Complutense University of Madrid, Pza. Ramón Y Cajal, s/n, Ciudad Universitaria, 28040 Madrid, Spain; 2 Laboratory of Cognitive & Computational Neuroscience, Complutense and Polytechnic Universities of Madrid Joint Laboratory, Centre for Biomedical Technology, Pozuelo de Alarcón, Spain; 3 Statistics & Operations Research Department, Faculty of Medicine, Complutense University of Madrid, Madrid, Spain; 4 Electrical Engineering & Bioengineering Group (EE&B), Industrial Engineering Department, University of La Laguna, Tenerife, Spain

**Keywords:** ApoE4, Cognitive deterioration, Cognitive phenotype, Healthy aging, Preclinical and prodromal Alzheimer’s disease, Amyloid markers

## Abstract

Whether the deleterious effects of APOE4 are restricted to the Alzheimer’s disease (AD) spectrum or cause cognitive impairment irrespectively of the development of AD is still a matter of debate, and the focus of this study. Our analyses included APOE4 genotype, neuropsychological variables, amyloid-βeta (Aβ) and Tau markers, FDG-PET values, and hippocampal volumetry data derived from the healthy controls sample of the ADNI database. We formed 4 groups of equal size (*n* = 30) based on APOE4 carriage and amyloid-PET status. Baseline and follow-up (i.e., 48 months post-baseline) results indicated that Aβ-positivity was the most important factor to explain poorer cognitive performance, while APOE4 only exerted a significant effect in Aβ-positive subjects. Additionally, multiple regression analyses evidenced that, within the Aβ-positive sample, hippocampal volumetry explained most of the variability in cognitive performance for APOE4 carriers. These findings represent a strong support for the so-called *preclinical/prodromal hypothesis*, which states that the reported differences in cognitive performance between healthy carriers and non-carriers are mainly due to the APOE4’s capability to increase the risk of AD. Moreover, our results reinforce the notion that a synergistic interaction of Aβ and APOE4 elicits a neurodegenerative process in the hippocampus that might be the main cause of impaired cognitive performance.

## Introduction

The noxious effects that the ɛ4 allele of the apolipoprotein E gene (i.e., APOE4) exerts on brain functioning have been associated with impaired cognitive performance and an increased risk of late-onset Alzheimer’s disease (AD) [1, 2]. Interestingly, however, the idea that APOE4’s detrimental influence on cognition might be limited to the AD spectrum is a matter of strong debate. Some studies reported that APOE4 carriage might predict the appearance of cognitive deficits along the lifespan, even in non-demented populations (see for example [3, 4]). This line of evidence led to the proposal of the *cognitive phenotype hypothesis*, which assumes that the ɛ4 allele induces a neural damage that accumulates over time, leading to cognitive impairment irrespective of the development of AD [5, 6].

On the other hand, the linkage between APOE4 and late-onset AD is one of the major foundations of the *preclinical/prodromal hypothesis*, which poses that any difference in cognitive performance that emerges between healthy APOE4 carriers and non-carriers would be secondary to the ɛ4 allele’s capability to increase the risk of AD [7]. This perspective was supported by retrospective studies indicating that the impaired performance observed in initially considered cognitively healthy APOE4 carriers was indeed explained by the inadvertent inclusion of preclinical or prodromal AD cases. When subjects progressing to AD were eliminated from the baseline analyses, APOE4 effects tended to disappear [8–10]. More recently, Foster and coworkers [11] reported that, when the statistical methods removed the effects due to the inclusion of MCIs (considering them as prodromal AD cases), the significant influence of APOE4 vanished. A recent investigation by our group also reinforced the preclinical/prodromal assumption [12]. Inspired by Foster et al.’s study [11], we evaluated APOE4’s effects on cognitive performance in a sample of healthy aged controls and observed that APOE4 carriers exhibited a significantly poorer performance on several cognitive domains, but such effect was explained by the group with a higher risk of presenting cognitive deterioration. Notably, in these investigations, the amyloid-βeta (Aβ) status of the participants was not taken into consideration. This is a crucial fact since, according to the criteria of the National Institute on Aging and the Alzheimer’s Association (NIA-AA) workgroup, the presence of a positive marker of amyloidosis is sufficient to define the stage 1 of preclinical AD in otherwise cognitively healthy population [13].

Assuming the crucial role of biomarkers to characterize preclinical AD cases and trying to shed light on the debate between the *cognitive phenotype* and the *preclinical/prodromal hypotheses*, we combined here APOE4 genotype and neuropsychological assessments with Aβ markers derived from PET scanning and CSF analysis, as well as with FDG-PET, hippocampal volumetry, and Tau markers. Such information was obtained from the healthy controls cohort of the ADNI database. The goal of this investigation was twofold. We first aimed to determine whether APOE4’s pernicious effects on cognitive performance are restricted to Aβ-positive individuals as some recent investigations suggested [14], which would provide a genuine validation for the *preclinical/prodromal hypothesis*. Secondly, we planned to assess whether APOE4 genotype affects cognition directly or via an associated neurodegenerative process such as hippocampal atrophy (see for example [15]).

## Materials and methods

Data used in the preparation of this article were obtained from the Alzheimer’s Disease Neuroimaging Initiative (ADNI) database (adni.loni.usc.edu). The ADNI was launched in 2003 as a public–private partnership, led by Principal Investigator Michael W. Weiner.

### Participants

We first downloaded from the ADNI website all the data and information regarding our variables of interest (APOE4, neuropsychological measures, Aβ, FDG-PET, Tau/pTau, and hippocampal volumetry). We performed an initial cleaning of that database by selecting only the healthy controls at the initial evaluation timepoint (i.e., baseline) whose neuropsychological, neuroimaging, and biomarker assessments were performed not more than 1 year and a half apart. Based on our experimental questions, we kept in the database only those healthy controls for whom information on both APOE4 and amyloid-PET was available, leaving an *initial clean cohort* of 216 participants (120 females; mean age: 72.80 ± 6.56). Importantly, the ADNI database offers information on several variables of interest not only at baseline evaluation but also at different timepoints of follow-up. Follow-up information of cognitive performance was used here to add further evidence on the potential individual and interaction effects of APOE genotype and Aβ burden (see the “Results” section below for more details about the specific timepoint selected).

All participants signed an informed consent, and the study was approved by the local institutional review board at each participating site.

### Neuropsychological assessment

Regarding the neuropsychological and cognitive performance evaluation, the following measures were included: (i) Alzheimer’s Disease Assessment Scale–Cognitive Subscale (ADAS11; [16]); (ii) MiniMental State Examination (MMSE; [17]); (iii) Montreal Cognitive Assessment (MoCA; [18]); (iv) Rey’s Auditory Verbal Learning Test (RAVLT), from which we included measures of immediate recall (RAVLT_IR), learning (RAVLT_LT), and rate of forgetting (RAVLT_RoF; [19]); (v) logical memory delayed recall (LMDR; [20]); (vi) Trail Making Test (TMT; [21]), including in the analyses the time of completion of parts A (TMTA-Time) and B (TMTB-Time); (vii) verbal semantic fluency (SF; [22]); and (viii) total score of the Boston Naming Test (BNT, [23]).

### Educational attainment

Considering previous investigations that highlighted the role of educational attainment as a sensitive estimate of cognitive reserve [24], this proxy was measured as years of formal education.

### APOE genotyping

Ethylenediaminetetraacetic acid (EDTA) blood samples were obtained from every participant, and the ADNI Biomarker Core performed the APOE genotyping on them using TaqMan assays, as previously described [25]. According to the presence or absence of the ɛ4 allele, subjects were classified as APOE4 + or APOE4 − .

### PET imaging

The detailed protocols of ADNI PET data acquisition and processing are available at: http://adni.loni.usc.edu/data-samples/pet/. Briefly, the standardized uptake values (SUVs) for ^18^F-AV-45 (florbetapir) were measured bilaterally at the cortical level in frontal, anterior and posterior cingulate, lateral parietal, and lateral temporal cortex. In order to normalize those values, the SUV for the whole cerebellum was also calculated. The mean normalized uptake value ratios (mcSUVRs) were thus obtained by dividing the averaged SUVs of the cortical regions by the SUV of the cerebellum. Amyloid positivity was determined by applying a cut-off value of 1.11 to the mcSUVRs measurement (for further details see for example [26]). Thus, subjects with mcSUVRs above the cut-off were considered amyloid-PET positive (Aβ +), while subjects with values below the cut-off were considered amyloid-PET negative (Aβ −).

The FDG-PET measure used here corresponds to the sum of mean glucose metabolism measured in bilateral angular, temporal, and posterior cingulate regions (for a review of this procedure see [27]).

### CSF markers

Participants at each ADNI site underwent a lumbar puncture the morning after an overnight fast. Then, the extracted CSF samples were shipped overnight to the ADNI Biomarker Core where aliquots (0.5 ml) from the samples were prepared and stored in bar-code-labeled polypropylene vials at − 80 °C. Total Tau (CSF_TTau), Aβ42 (CSF_Aβ42), and phosphorylated Tau (CSF_pTau) were assayed from the prepared aliquots using the multiplex xMAP Luminex platform (Luminex Corp., Austin, TX) with Innogenetics immunoassay kit–based reagents (INNO-BIA AlzBio3; Ghent, Belgium; for research use-only reagents) [25, 28].

### MRI

Participants at each ADNI site were scanned in a 1.5 or 3.0 T structural brain MRI machines using a standardized protocol [29]. All structural data passed quality control and were pre-processed, and volumetric measures of regions across the whole brain were obtained by using FreeSurfer v. 5.1. (analysis performed at UCSF). Specifically, we were interested in the volume values of left and right hippocampi, which could be deemed as indices of neural degeneration. Volume values for bilateral hippocampi were normalized by dividing this measure by the overall intracranial volume (ICV). These normalized values for left (LH_ICV) and right (RH_ICV) hippocampus were included in our statistical analyses.

### Statistical analyses

The main aim of this study was to investigate the relationship between cognitive performance as measured by means of neuropsychological evaluation, Aβ burden as measured by means of amyloid-PET, and APOE genotype. However, some other factors such as sex, age, years of education, hippocampal volumes, FDG_PET values, and Aβ42 or Tau-related levels in CSF were considered as well. Parametric (ANOVA) and non-parametric (Kruskal–Wallis) tests were employed for the group comparisons of quantitative variables. Before the between-group comparison, conformity with the assumptions of ANOVA was ensured by applying Shapiro–Wilk test for assessing normality of the variables, and Levene’s test for evaluating the homoscedasticity. The relationship between qualitative variables was assessed by using chi-square test of homogeneity. Overall, quantitative variables were described as means and standard deviation, and qualitative variables were described as absolute frequency and percentages, unless stated otherwise.

The combined effects of APOE genotype and Aβ burden on cognitive tests were assessed through a series of ANOVAs with two factors, APOE4 status (i.e., APOE4 + vs. APOE4 −) and amyloid-PET status (i.e., Aβ + vs Aβ −). Post hoc comparisons were evaluated and corrected for multiple comparisons using Bonferroni’s test. Once ANOVA models were accomplished, the relationship between two quantitative variables was assessed using Pearson’s correlation for normally distributed variables or Spearman’s correlation coefficient for variables that failed to meet a normal distribution. Multiple regression models using a forward selection method were employed to further investigate the relationship between cognitive scores and other factors of interest (i.e., age, hippocampal volumes, APOE4, Aβ and Tau-related markers). Finally, a series of repeated measures ANOVAs were used to compare the differences between cognitive scores at baseline and after a 48-month follow-up period.

SPSS v. 25 was used for all statistical analyses. Results were considered statistically significant when showing a *p*-value < 0.05. Regarding the graphical representation of results, the MedCalc 19.7 software was used to generate the graphs and the CorelDraw v. 2019 was applied to improve the aesthetical appearance of those graphs (i.e., color, thickness, and size of lines and dots, alignment of graphs, etc.) as well as to create the figure summarizing the main findings (Fig. 2).

## Results

### Amyloid positivity and APOE genotype: preliminary analyses

As it was previously described in the “Materials and methods” section, our *initial clean cohort* consisted of 216 healthy control subjects. According to the study design, the sample was classified into four groups: 117 APOE4 − _Aβ − cases, 31 APOE4 + _Aβ − cases, 38 APOE4 − _Aβ + cases, and 30 APOE4 + _Aβ + cases. While testing between-group differences, a significant effect of age was found, with both Aβ + groups showing significantly older ages than the Aβ − groups (all *p*-values < 0.001). Additionally, a remarkable unbalanced sample size was observed when the four groups were compared. In order to avoid a potential spurious effect of age and considering that the ANOVA F-tests are more robust when group sizes are equal, we decided to refine our four groups by setting an identical number of subjects in all of them. Since the APOE4 + _Aβ + group was the smallest, formed by 30 individuals, we pseudo-randomly selected 30 subjects within the other three groups with one condition: the resulting groups should not show significant differences in terms of age (i.e., *p*-values > 0.05). Group differences in terms of sex distribution and years of education were also avoided. Table 1 displays the more important demographic information of the resulting 4 groups.

**Table 1:** Demographic information at baseline and 48-month timepoints. Means and standard deviations (in parentheses) are detailed for quantitative variables, while gender data are represented by means of absolute frequency ratios (male/female) and percentage of male/female individuals (in parentheses). Importantly, no significant between-group differences were found at any of the evaluation timepoints

	APOE4 − _Aβ-	APOE4 + _Aβ-	APOE4 − _Aβ +	APOE4 + _Aβ +
*Age*				
Baseline	71.570 *(7.049)*	70.013 *(5.071)*	74.160 *(4.519)*	73.660 *(6.708)*
48-months	74.150 *(5.901)*	71.259 *(5.541)*	74.841 *(4.078)*	73.980 *(5.907)*
*Education*				
Baseline	16.833 *(2.214)*	15.866 *(2.515)*	15.400 *(2.268)*	16.533 *(2.501)*
48-months	16.72 *(2.270)*	15.76 *(2.538)*	15.00 *(1.904)*	16.27 *(3.011)*
*Gender (male/female)*				
Baseline	16/14 *(53.3/46.7)*	10/20 *(33.3/66.7)*	9/21 *(30.0/70.0)*	11/19 *(36.7/63.3)*
48-months	12/6 *(66.7/33.3)*	6/11 *(35.3/64.7)*	4/13 *(23.5/76.5)*	8/7 *(53.3/46.7)*

### Baseline evaluation: analysis of group effects on cognitive functioning

**Fig. 1:**
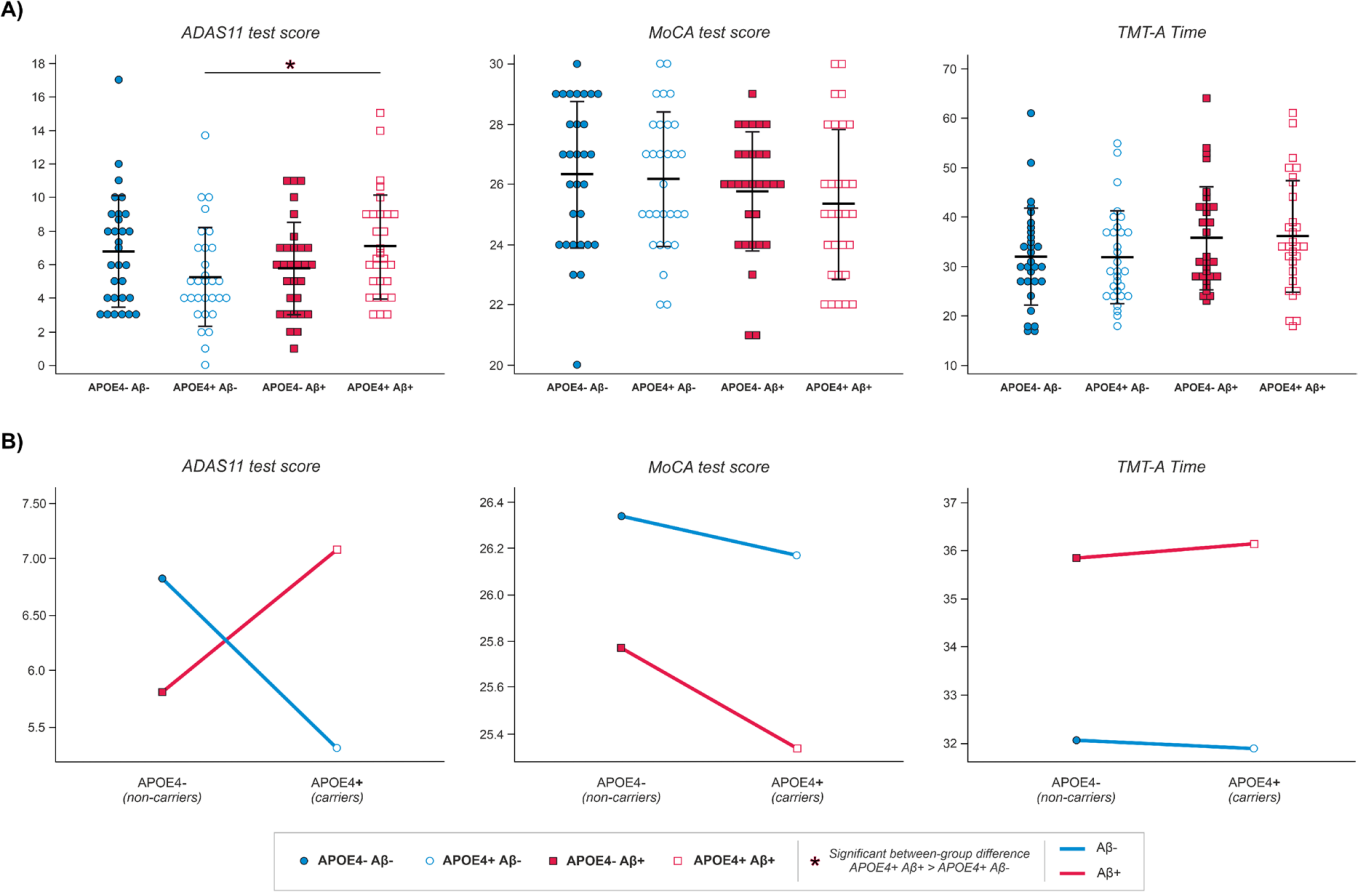
Depiction of distribution across groups and effects of APOE genotype and amyloid-PET on the neuropsychological variables selected (i.e., scores of ADAS-11 and MoCA tests, and total time for TMT-A) at baseline evaluation timepoint. (**A**) Distribution of scores in the neuropsychological variables of interest; black lines show mean and standard deviation for each group and variable. Significant between-group differences are marked with an *. (**B**) Interaction plots: means of cognitive performance in the three neuropsychological variables selected displayed according to the combination of APOE genotype and amyloid-PET effects

In a second step of the statistical analyses, we investigated the combined effects of APOE4 and amyloid_PET status on cognitive functioning. Overall, the main effect of APOE4 factor failed to show any statistically significant effect (all *p*-values > 0.255). On the contrary, the main effect of amyloid_PET status was significant for TMTA_Time and MoCA. Mean values of TMTA_Time were significantly higher (*p* = 0.016), while mean values of MoCA scores were significantly lower (*p* = 0.048) in Aβ + subjects, both indicating a poorer cognitive performance in this population compared to the Aβ − subjects (see Fig. 1).

Interestingly, ADAS11 results demonstrated a significant interaction between APOE4 and amyloid_PET status (*p* = 0.013). Post hoc comparisons revealed that the APOE4 + _Aβ − subgroup exhibited significantly lower scores on ADAS11 than the APOE4 + _Aβ + subgroup (*p* = 0.024). Considering that lower scores on the ADAS11 indicate a better cognitive status, these results suggested a more preserved functioning in the APOE4 + _Aβ − group. No statistically significant differences emerged when comparing other group-combinations (all *p*-values > 0.199). (See Fig. 1 for a depiction of the described effects.)

### Baseline evaluation: multiple regression analyses of cognitive scores

An analysis of the data, depicted in panel B of Fig. 1, suggests that the effects of APOE4 expression on cognition were negligible when this specific genotype was not accompanied by Aβ-positivity. A crucial question posed by these results is whether some additional factors of interest might further explain the observed variability on cognitive performance within the Aβ + cases. With this aim, a series of exploratory Pearson’s or Spearman’s correlation coefficients (depending on normality of the variables’ distribution) were performed to detect factors that might be considered potential predictors. As displayed in Table 2, four variables (LH_ICV, RH_ICV, CSF_Aβ42, and age) showed a significant relationship with the three previously selected cognitive tests (ADAS11, MoCA, and TMTA_Time; all *p*-values ≤ 0.003). The rest of the factors (i.e., years of education, FDG-PET, and Tau-related levels) failed to show any significant correlation with cognitive performance.

**Table 2:**
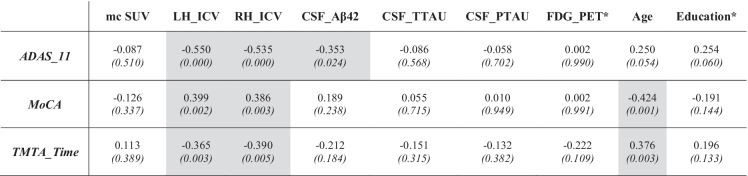
Details regarding the correlations performed between cognitive tests scores and the neuroimaging, biomarker, and demographic variables for the Aβ + group. Pearson’s correlations were applied in all but two variables, whose distribution was not normal (i.e., FDG-PET and Education). For variables not following normal distributions (marked in this table with a *), Spearman’s correlations were applied. Pearson’s/Spearman’s correlation coefficient and *p*-values (in parentheses and italic font) are detailed. Cells with gray background indicate the statistically significant results

Once candidate variables were identified by correlation analysis, they were all submitted to a series of multivariate regression analyses by using forward selection in order to pick those that should be included in the final models. Importantly, the multivariate regression models were first calculated for the whole sample of Aβ + cases and then separately in APOE4 − _Aβ + and APOE4 + _Aβ + groups to investigate the specific contribution of APOE4 carrier status within the Aβ + individuals.

**Fig. 2:**
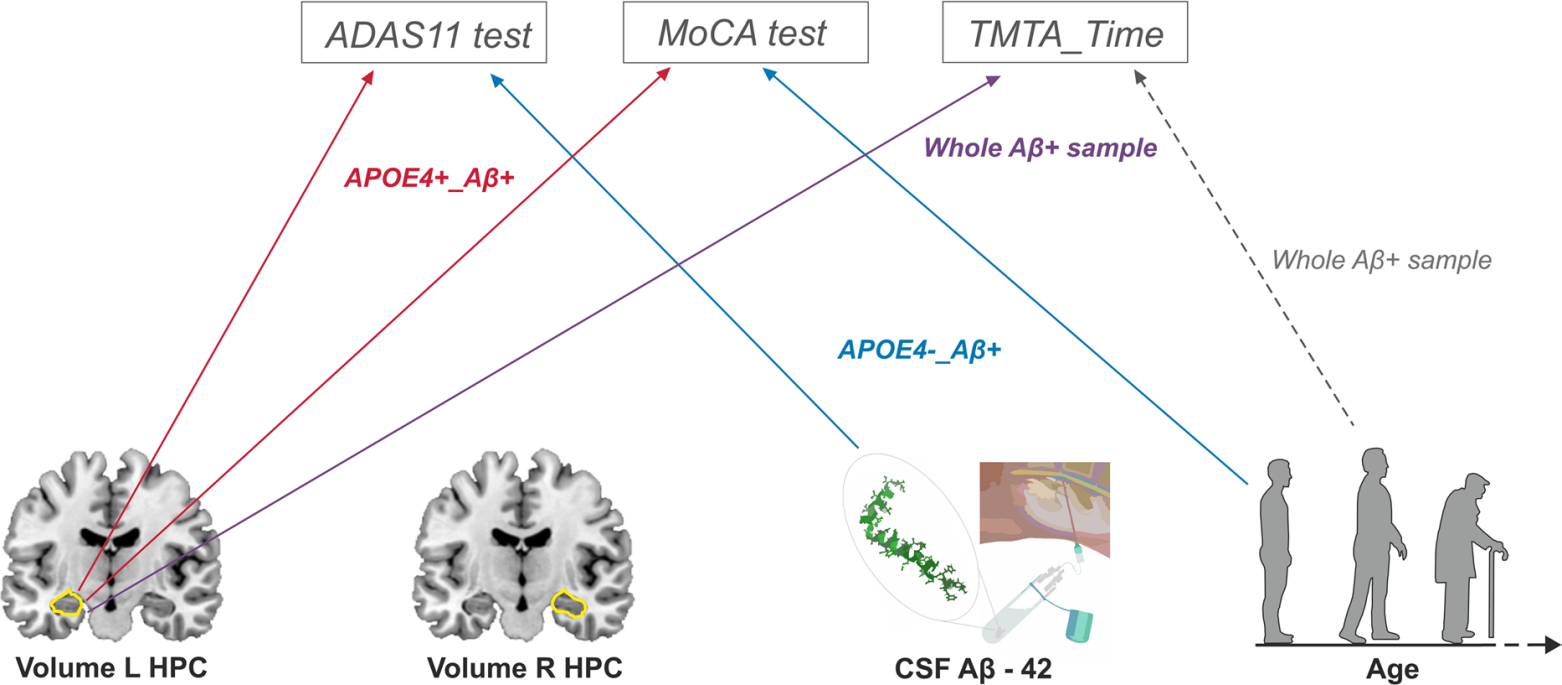
Summary of results of the multiple regression analyses of cognitive scores for Aβ + subjects at baseline evaluation timepoint (although the general findings remain the same when taking into account the follow-up data). At the top of the image, the selected cognitive tests are displayed in gray rectangles (ADAS11, MoCA, and TMTA_Time tests). In the bottom, the four factors showing a significant relationship with the aforementioned cognitive tests can be found depicted in a schematic way (from left to right): volume of left hippocampus (HPC), volume of right HPC, CSF_Aβ42, and Age. Lines represent which factors (at the bottom) better explained the variability of which cognitive test (at the top), being color-coded depending on the sample/subsample for which this relationship was found: red for APOE4 + _Aβ + individuals, blue for APOE4 − _Aβ + subjects, violet and gray for the whole Aβ + sample, with the gray dotted line representing a non-significant trend

Table 3 displays the final multivariate regression models. For ADAS11, LH_ICV was the first variable selected by the model, while CSF_Aβ42 was next when the whole sample of Aβ + cases was considered. Within APOE4 + _Aβ + group, only LH_ICV was capable of significantly explaining the variability of ADAS11, with lower volumes predicting higher scores, thus indicating a worse cognitive status. Within the APOE4 − _Aβ + cases, CSF_Aβ42 was the only variable significantly associated with ADAS11, suggesting that lower Aβ42 levels predicted a worse cognitive performance. In the case of the MoCA test, LH_ICV was again the top variable suggested by the model, while age was second when the whole sample of Aβ + cases was considered. Mirroring ADAS11, when the APOE4 + _Aβ + group was analyzed, LH_ICV values were sufficient to explain the variability of MoCA scores, with higher volumes predicting better performance. On the other hand, age was the only variable that significantly predicted the variability of MoCA scores within the APOE4 − _Aβ + group, with older subjects exhibiting a worse cognitive status. The pattern observed for TMTA_Time scores was a bit different, as age exhibited a weak capability to explain test scores that failed to reach statistical significance, and only LH_ICV was significant in the whole sample. These variables failed to show a significant influence when groups were analyzed separately. (See Fig. 2 for a summary of these results.)

**Table 3:** Results from the multivariate regression model. Regression coefficient, respective *p*-values (in parentheses), and Pearson’s correlation coefficient (*R*) are detailed for the significant results only

	Age	LH_ICV	CSF_Aβ42	Constant	*R*
*ADAS11*					
Aβ +	–	− 4209.273 *(0.004)*	− 0.003 *(0.035)*	18.991	0.565
APOE4 + _Aβ +	–	− 5828.712 *(0.009)*	–	21.718	0.581
APOE4 − _Aβ +	–	− 2876.762 *(0.090)*	− 0.004 *(0.022)*	16.195	0.595
*MoCA*					
Aβ +	− 0.126 *(0.020)*	2034.181 *(0.043)*	–	29.851	0.527
APOE4 + _Aβ +	–	3301.354 *(0.045)*	–	17.323	0.464
APOE4 − _Aβ +	− 0.196 *(0.018)*	2015.429 *(0.077)*	–	35.094	0.625
*TMTA_Time*					
Aβ +	0.490 *(0.077)*	− 12,080.789 *(0.005)*	–	20.966	0.427
APOE4 + _Aβ +	–	–	–	–	–
APOE4 − _Aβ +	–	–	–	–	–

At this point it is important to note that a correlation analysis, identical to the one displayed in Table 2, was performed within the Aβ − sample. Interestingly, only a negative correlation between age and MoCA test scores was found to be statistically significant (*p* = 0.006).

### Follow-up evaluation: evolution and group effects on cognitive functioning

As previously stated, the ADNI database offers follow-up information on cognitive scores at different post-baseline evaluation timepoints. For the subsample selected in the present report, we found that the information contained in the 48-month post-baseline evaluation database was more complete in terms of number of participants (APOE4 − _Aβ − *n* = 18, APOE4 + _Aβ − *n* = 17, APOE4 − _Aβ + *n* = 17, APOE4 + _Aβ + *n* = 15). Importantly, between-group differences in terms of age, gender, and education were also not significant at this follow-up evaluation (*p* > 0.05. Details of demographic information at the 48-month follow-up timepoint can be found in Table 1). Hence, we decided to compare the cognitive performance registered at the 48-month evaluation timepoint with the scores obtained at baseline. Such comparison was restricted to the three cognitive variables that exhibited significant differences at baseline due to APOE genotype and Aβ status effects (i.e., ADAS-11, MoCA, and TMTA_Time), and had two main goals: (1) to determine whether cognitive scores showed a significant variation or evolution within each group across time and (2) to determine whether between-group differences detected at baseline evaluation remained significant after a 48-month follow-up period.

Regarding ADAS-11, we failed to find statistically significant differences (all *p*-values > 0.154) when baseline and 48-month scores were compared within each group using a repeated-measures ANOVA. Notably, when the between-group comparison was performed, we found that the significant interaction between APOE4 and amyloid_PET status observed at baseline disappeared after the 48-month follow-up period (*p* = 0.330). However, the main effect of Aβ status remained significant and, mirroring baseline results, the APOE4 + _Aβ − group exhibited significantly lower scores than the APOE4 + _Aβ + group (*p* = 0.028), indicating that the latter still showed the worst cognitive status.

Similarly, when MoCA scores at baseline were compared to those obtained after the 48-month follow-up period, no statistically significant differences were detected within each group (all *p*-values > 0.416). Mirroring ADAS-11 results, MoCA values at 48-month evaluation showed a maintenance of the main effect of amyloid_PET status (*p* = 0.05), indicating that APOE4 + _Aβ − group exhibited significantly higher scores than the APOE4 + _Aβ + group (*p* = 0.007), thus implying that the latter showed again the worst cognitive status.

**Fig. 3:**
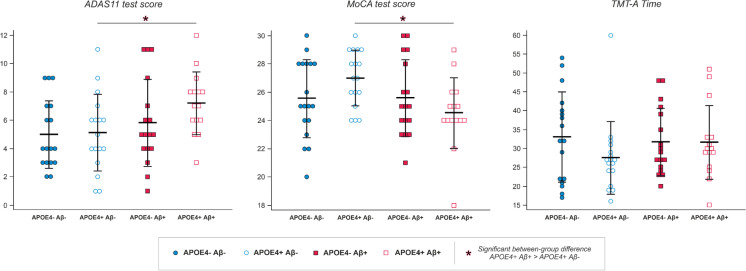
Distribution across groups of the scores in the neuropsychological variables selected (i.e., ADAS-11, MoCA, TMT-A) at the 48-month follow-up evaluation timepoint; black lines show mean and standard deviation for each group and variable. Significant between-group differences are marked with an *, generally showing that APOE4 + _Aβ + individuals are the ones performing the worst in these cognitive tests

Finally, within-group comparison of TMTA_Time scores at baseline and after 48 months failed to show statistically significant differences (all *p*-values > 0.070). In this case, the main effect of amyloid_PET status observed at baseline evaluation disappeared at the 48-month follow-up evaluation (*p* = 0.581). (See Fig. 3 for a depiction of the follow-up results.)

## Discussion

As explained in the “Introduction” section, the goal of this study was twofold. First, we tried to elucidate if the assumed effects of APOE4 on cognition were restricted to Aβ + individuals, and therefore to those that might be considered within the AD-continuum. Second, we aimed at determining if such potential influence of the ɛ4 allele might be mediated by a neurodegenerative process, specifically explored here by looking at possible reductions in hippocampal volumes. As a matter of fact, our findings supported both assumptions, and we discuss them in detail in the following paragraphs.

Relatively early studies reported that APOE4 exerted a particular effect on memory functioning within healthy elders (see, for instance, [30]). Further, meta-analyses by Small et al. [31] and, later on, Wisdom et al. [32] revealed that APOE4 influences several cognitive domains, including episodic memory but also global cognitive functioning, executive functioning, and perceptual skills. However, as Flowers and Rebeck [33] pointed out, the direct implication of APOE4 in the increase of Aβ deposition makes it difficult to establish a clear-cut determination of their individual and interaction effects. In fact, these authors suggested that studies in middle-aged or even older subjects should contemplate the influence of both Aβ and APOE4 genotype on brain structures. For example, investigations such as those reported by Caselli and coworkers [34, 35] or Villemagne and collaborators [36] evidenced independent effects of both factors but their interaction was not explored. Further, Lim et al. [37] claimed that cognitive decline only appeared in healthy controls that were APOE4 carriers but their study was restricted to Aβ + cases and, consequently, a potential interaction could not be fully assessed. On the contrary, studies such as those presented by Sperling et al. [38] or Roe et al. [39] demonstrated that Aβ levels seemed to be sufficient to explain the differences in episodic memory or global cognitive performance observed in healthy aged controls.

Interestingly, the perspective in this field changed when the potential interaction between APOE and Aβ status was systematically taken into consideration. For instance, Kantarci et al. [40] reported that the association between impaired cognition and Aβ-burden was more evident in APOE4 + cognitively normal elders. In addition, these authors found a significant interaction between APOE and Aβ effects in several cognitive domains, although the statistical analyses failed to find an isolated influence of APOE. Mormino et al. [41] found very similar results, with APOE4 + _Aβ + controls showing a progressive deterioration in episodic memory, while other combinations of factors did not yield any relevant effects. Notably, Donohue and coworkers [42] also reported that controls carrying APOE4 exhibited a more pronounced cognitive decline but claimed that Aβ levels were the crucial factor to explain that process. This reasoning thread was strongly reinforced by Lim et al.’s [14] investigation. Briefly, these authors investigated whether the age-associated memory decline was greater in APOE4 carriers than in non-carriers, exploring, as well, the potential interaction between genetics and Aβ-burden. Their findings suggested that the cognitive decline started earlier and was more pronounced in APOE4 + _Aβ + cases but, more importantly, confirmed the hypothesis that, in absence of Aβ-positivity, APOE4 was not associated with an age-related memory decline. This line of evidence was confirmed by data derived from animal models which also suggested that APOE4 has no toxic effects in the absence of Aβ. For example, Griffin et al. [43] used humanized transgenic nematodes to facilitate neuronal modeling of Aβ co-expression in the context of distinct human APOE alleles. Their results proved that APOE isoforms had no functional impact in the absence of Aβ co-expression.

Our current results confirmed the tendencies observed in the above cited investigations. First, we failed to find an isolated effect of APOE4 on cognitive tests. Then, we found that it was Aβ-positivity the factor explaining worse performance among healthy subjects in MoCA and TMTA_Time, without any influence of APOE4 carrier status. Finally, an interaction between Aβ-burden and APOE genotype was observed in ADAS11, with APOE4 + _Aβ + subjects exhibiting the poorest performance. Thus, APOE4 only affected ADAS11 scores when accompanied by Aβ-positivity.

Follow-up results reinforced this tendency. Within-group comparisons failed to find significant differences between baseline and follow-up scores, although MoCA values appeared slightly reduced in the APOE4 + _Aβ + group. Consequently, we cannot justify a “progression” of cognitive deterioration, as described by Mormino et al. [41]. However, between-group comparisons demonstrated that the APOE4 + _Aβ + group remained as the one with the worst cognitive performance after the follow-up period, both for ADAS11 and MoCA tests. ADAS11 results are of particular interest since the significant interaction between Aβ-burden and APOE genotype observed at baseline disappeared, and Aβ effects emerged as the most important factor to explain the observed differences at the 48-month evaluation. Hence, our baseline and follow-up findings, jointly with previously described evidence, suggest that APOE4 might exert a limited deleterious effect that is especially exacerbated by the presence of elevated Aβ levels, only then producing a detectable cognitive impairment (see below).

The second goal of our study was to assess whether the presumed APOE4 effects on cognition were mediated by a neurodegenerative process such as hippocampal atrophy. Indeed, our results indicated that LH_ICV scores were sufficient to explain worse performance in ADAS11 and MoCA tests within APOE4 + _Aβ + cases, as compared with APOE4 − _Aβ + cases. These evidences somewhat paralleled those obtained in Li and collaborators’ [27] investigation, whose main goal was to assess the combined effect of APOE4 and Aβ load on brain cortical thickness, evaluating their impact on cognitive functioning as well. Li et al. [27] reported that APOE4 carriers with significant Aβ pathology (i.e., subjects that would correspond with our APOE4 + _Aβ + group) exhibited impaired cognitive scores when compared to all other groups. Notably, ɛ4 status was associated with reductions in hippocampal volumes and in cortical thickness in limbic regions that, in turn, correlated with impaired memory and general cognition [27].

Although some controversy exists in samples of healthy controls (see [44]), the evidence of a link between APOE4, hippocampal atrophy, and impaired cognition seems robust. On the one hand, the medial temporal lobe (MTL) has been considered one of the brain regions more severely affected by APOE4’s detrimental effects, with carriers tending to exhibit a greater rate of atrophy in follow-up studies [45, 46]. On the other hand, markers of MTL atrophy parallel cognitive symptomatology within the AD spectrum [47]. According to Jack et al.’s [47] perspective, the role of Aβ deposition might be more problematic since biomarkers tend to reach a plateau before the appearance of both atrophy on MRI and cognitive symptoms. However, Mormino et al. [48] demonstrated the influence of Aβ load on both hippocampal volumes and cognition in a sample composed of healthy controls and Aβ + MCI cases. Using linear regression models, these authors observed that Aβ load explained hippocampal-volumes’ variability and, in turn, hippocampal volume was the main predictor of cognitive performance. In their ulterior study, Mormino and coworkers [41] affirmed that the existence of a significant interaction between Aβ and APOE4 indicates that these factors do not reflect redundant information. Authors proposed that the combination of APOE4 and high Aβ levels might result in higher quantities of underlying pathology, such as neurofibrillary tangles, thus contributing to a neurodegenerative process that initiates in the MTL [49]. Similarly, a recent investigation by Ge and colleagues [50] claimed a so-called synergistic interaction between Aβ and APOE4 genotype, based on the fact that Aβ + subjects carrying the ɛ4 allele exhibited an accelerated cognitive decline that was accompanied by hippocampal degeneration.

Our results show a clear support to Mormino et al.’s [41] and Ge et al.’s [50] findings and proposals. The main conclusion of our linear regression modeling was that hippocampal volumes were the main predictors of cognitive performance, especially in APOE4 carriers. Interestingly, the variability of cognitive scores within APOE4 − _Aβ + cases was related to other factors such as age or CSF_Aβ42 levels. Age is a well-known factor influencing cognitive performance that also interacts with APOE genotype to produce a variety of outcomes (for a review, see [12]). In addition, older ages are associated with an augmented Aβ-burden in healthy controls that increase the risk of cognitive deterioration [51]. Certainly, Aβ-deposition is measurable not only by means of PET estimates but also by means of Aβ42 levels in the CSF. Both markers exhibit a good correlation, with low CSF_Aβ42 levels being associated with an increased cognitive impairment in healthy controls [52], as it was observed in our sample.

Perhaps a more noticeable evidence derived from our results is the lack of influence of other potential AD markers such as FDG-PET or CSF_TTau and CSF_pTau levels. This finding is remarkable for Tau markers as they have been consistently associated in previous reports with cognitive manifestations, especially in MCI and AD cases [53, 54]. Previous reports studying the effects of different biomarkers on the pathophysiology and disease progression along the AD spectrum using the ADNI database did show an important effect of CSF_Tau variables (TTau, pTau), although the pathophysiological timeline of these biomarkers was not so clear. Earlier reports concluded that Aβ_42_ changes precede Tau changes ([55, 56]). However, some more recent reports argued that CSF_Tau is crucial for predicting cognitive decline and for the diagnosis of early-MCI, and that its alterations appear to begin even earlier than hippocampal atrophy or cognitive decline signs, especially when looking at APOE4 carriers, who showed elevated values of CSF_Tau already in the prodromal AD stage [57–60]. Despite these evidences of CSF_Tau variables as key biomarkers in AD progression, their effects on cognition seemed to be observed only when considering MCI and AD subjects or when including all diagnoses of the ADNI dataset [57, 59, 61, 62], and not when looking only at the normal-cognition controls, as it is the case in our report. Recent reviews that included other datasets or were focused on completely different cohorts support this same view [52, 63–68]. Therefore, our findings seem to support a framework in which Aβ-burden is not the only, but is probably the main, factor responsible for cognitive decline in very early stages of the AD spectrum (see [26]).

## Conclusions and limitations

The current study presents a main limitation. Our intent to obtain balanced groups in terms of age, sex, and years of formal education had positive consequences for the robustness of the statistical analyses but reduced the sample size to a total of 120 cases. Despite these limitations, we believe the present study still offers quite relevant information on the neurobiological basis of cognitive variations in otherwise healthy control subjects. Results demonstrated that Aβ-positivity is the main cause of a reduced cognitive performance in this sample. APOE4 failed to show a significant individual effect, and only produced a relevant influence in Aβ + cases (so-considered preclinical AD). Thus, evidence observed here represents a strong support for the *preclinical/prodromal hypothesis*. In addition, we confirmed that Aβ and APOE4 effects converge to produce a neurodegenerative process in the MTL that seems to be the ultimate cause of the impaired performance observed in our sample.

## Data Availability

Data is available at: adni.loni.usc.edu, and can be accessed following the regulations by ADNI.

## Code availability

Code regarding the ADNI part of the analysis is available under ADNI regulations. The authors’ code of analysis for the current study will be made available upon request (always after consultation with the corresponding personnel from ADNI).
